# A TDMA-Based MAC Protocol for Mitigating Mobility-Caused Packet Collisions in Vehicular Ad Hoc Networks

**DOI:** 10.3390/s22020643

**Published:** 2022-01-14

**Authors:** Muhammad Bilal Latif, Feng Liu, Kai Liu

**Affiliations:** 1 School of Electronics and Information Engineering, Beihang University, Beijing 100191, China; m.bilal.latif@gmail.com (M.B.L.); liuf@buaa.edu.cn (F.L.); 2 Hangzhou Innovation Institute, Beihang University, Hangzhou 310051, China

**Keywords:** vehicular ad hoc networks, medium access control, time division multiple access, merging collision mitigation, time-bounded message delivery

## Abstract

An autonomous driving environment poses a very stringent requirement for the timely delivery of safety messages in vehicular ad hoc networks (VANETs). Time division multiple access (TDMA)-based medium access control (MAC) protocols are considered a promising solution because of their time-bound message delivery. However, in the event of mobility-caused packet collisions, they may experience an unpredicted and extended delay in delivering messages, which can cause catastrophic accidents. To solve this problem, a distributed TDMA-based MAC protocol with mobility-caused collision mitigation (MCCM-MAC) is presented in this paper. The protocol uses a novel mechanism to detect merging collisions and mitigates them by avoiding subsequent access collisions. One vehicle in the merging collisions retains the time slot, and the others release the slot. The common neighboring vehicles can timely suggest a suitable new time slot for the vacating vehicles, which can avoid access collisions between their packet transmissions. A tie-breakup mechanism is employed to avoid further access collisions. Simulation results show that the proposed protocol reduces packet loss more than the existing methods. Consequently, the average delay between the successfully delivered periodic messages is also reduced.

## 1. Introduction

A medium access control (MAC) protocol with high performance is required for vehicular ad hoc networks (VANETs) to ensure road safety. In VANETs, each vehicle needs to send a periodic message every 100 ms to support the safety applications [1,2]. The timely delivery of messages is even more critical for autonomous driving environments [3,4,5]. On the one hand, contention-based MAC protocols such as IEEE 801.11p [6] cannot meet this stringent requirement [7]. On the other hand, contention-free MAC protocols, especially on the basis of time division multiple access (TDMA), can provide deterministic access to the channel for time-bound message delivery, thus becoming a research hotspot [8,9,10].

In a TDMA environment, the time is divided into periodic frames and the frames are placed into several time slots. Each vehicle in the VANET has a dedicated time slot in a frame for its data transmission. This has two-fold benefits: each vehicle is time-bound and obtains collision-free access to the channel in usual circumstances. However, two vehicles that are far apart from each other can be assigned the same time slot for scalability. At the time of slot allocation, the protocol should ensure that such time slot assignment does not cause communication interference among vehicles. However, due to vehicle mobility, two vehicles that possess the same time slot move closer, causing a packet collision between their transmissions, and both their packets are lost to their common neighboring vehicles. This phenomenon is known as the merging collision problem [8,10]. Merging collisions are one of the key factors which degrade the MAC performance for VANET [11,12]. However, solving it is a challenging task [8]. The existing protocols cannot completely eliminate the packet collisions caused by the merging collision scenarios [8].

VeMAC [13] is a popular distributed TDMA-based MAC protocol for VANET, which provides a reliable broadcasting mechanism for exchanging periodic messages. It also proposed a collision mitigation mechanism to avoid the merging collision scenario between vehicles moving in the opposite directions. This partitioned the TDMA frame into two disjoint fixed continuous portions. One portion is dedicated to being used for the vehicles moving towards the left direction and the second portion for the vehicles moving towards the right direction. With this scheme, two vehicles moving in opposite directions cannot have a common time slot, and thus a packet collision between them is avoided. However, the vehicles moving in the same direction can possess the same time slot and merging collisions between them can occur. In addition, if the vehicle density in one direction increases beyond the size of its dedicated partition, the VeMAC permits the vehicle to acquire a time slot from another partition which can also cause the merging collision between the two vehicles moving in opposite directions. Thereafter, MoMAC [14] extended the idea and proposed the further partitioning of each section according to the different road lanes, e.g., one subsection was dedicated for one lane. In this way, the merging collisions between vehicles moving in the same direction but in different lanes are mitigated. However, a merging collision may still occur between vehicles moving in the same lane. Similarly, several other works [4,15,16,17,18,19,20] have also focused on the frame partitioning technique to mitigate the merging collision problem.

In general, the frame partitioning technique cannot solve the merging collisions between the vehicles that can be assigned time slots in the same partition. Moreover, it limits the number of available time slots for the vehicles seeking a new time slot, which can cause more access collisions, thus degrading the MAC performance with the increase of vehicle density. To resolve these problems, this paper presents a distributed TDMA-based MAC protocol with mobility-caused collision mitigation (MCCM-MAC) and focuses on improving the performance of a distributed TDMA-based MAC protocol without depending upon the frame partitioning. We build on the VeMAC’s broadcasting mechanism and mitigate the packet collisions through novel mechanisms. Similar to VeMAC, each vehicle broadcasts the information of neighboring vehicles and can detect the loss of its slot on the basis of the received information from its neighbors. However, unlike VeMAC, with the proposed protocol, one of the vehicles in the merging collision retains its time slot, and other vehicles vacate their time slots. Therefore, the retaining vehicle does not need to compete for a new time slot and can successfully transmit in the next frame using its existing time slot, thereby avoiding further delay. If the number of vacating vehicles is only one, it can also successfully acquire a new time slot in the next frame as there will be no competitor. This is achieved through the proposed third parties slot-merge collision mechanism. However, in the case of multiple vacating vehicles, they have to compete for new time slots, which can cause further packet collisions. To mitigate this situation, the proposed protocol employs two novel resolution mechanisms, namely the slot suggestion mechanism and the tie-breakup mechanism, to avoid subsequent access collisions. The contributions of this paper are as follows:

- The paper highlights and formulates the extended delay problem, which the vehicles can experience in the event of a merging collision with a TDMA-based MAC protocol for VANET. This delay can be catastrophic, especially in an autonomous driving environment. We show that this delay is directly proportional to the number of collided packets soon after the merging collisions. Therefore, avoiding the subsequent packet collisions can restrict further prolonged delay.
- The proposed third parties slot-merge collision mechanism enables the vehicles to detect and mitigate the slot-merge collision of the neighboring vehicles. Consequently, one of the vehicles in the slot-merge collision retains the time slot, and others vacate the time slot. This eliminates the possibility of subsequent access collision in case of a merging collision between two vehicles.
- The proposed slot suggestion mechanism is employed to avoid the subsequent access collision between the vehicles that vacate their time slots due to the slot-merge collision. This can avoid the subsequent access collision in case of a merging collision between three vehicles.
- The proposed tie-breakup mechanism enables one of the vehicles in the access collisions to retain the time slot and others to vacate, thereby restricting further subsequent access collisions. This can avoid subsequent access collisions after an access collision.
- The extensive simulation results have been presented to show the effectiveness of the proposed protocol.

The remainder of the paper is organized as follows: Section 2 describes the related work. Section 3 introduces the necessary background and formulates the problem. Section 4 presents the proposed protocol. Section 5 details the simulation studies, and finally, Section 6 concludes the paper.

## 2. Related Work

Many TDMA-based MAC protocols have been proposed for VANETs. The survey works [8,9,21,22] encompass a considerable number of these protocols. One popular classification is based on the slot management process. It can be either controlled by a central entity [18,23,24,25,26] or managed by each vehicle on its own [13,14,16,20,27,28], thus categorizing the protocols into the centralized MAC protocols and distributed MAC protocols, respectively [21]. The centralized MAC protocols require either roadside units (RSU) or cluster heads (CH) to be in operation. On the other hand, the distributed MAC protocols do not require any centralized coordinators and thus are easier to deploy. However, solving the merging collisions is more challenging in distributed protocols due to the absence of any central coordinator for slot management. The focus of this work is the distributed TDMA MAC protocols for VANETs. Then, some of the protocols are based on purely the TDMA technique, e.g., [13,14,16,20,28], and some combine it with the contention-based approach to form a hybrid approach, e.g., [29,30,31,32,33]. Our work is based purely on the TDMA technique.

ADHOC-MAC [34] provides a reliable broadcasting mechanism for exchanging periodic messages in a TDMA fashion, which may be considered a pioneer TDMA-based MAC protocol for VANET. Based on this, VeMAC [13] improved the broadcasting mechanism further by defining the slot release prevention condition. Using the frame partitioning technique, VeMAC [13] and MoMAC [14] formed fixed-size partitions to reduce the packet collisions. However, this method resulted in the channel wastage problem under the unbalanced traffic conditions. Therefore, A-VeMAC [16], AODMAC [20] and SAMD [19] used adaptive frame partitioning, i.e., partition length is dynamically adjusted according to the vehicle density in the respective sections. Fine-grained MAC [4] used a dynamic beacon rate to address the slot shortage problem but used the same solution as MoMAC for the merging collision. None of the above work can mitigate merging collisions between vehicles in the same partition. Additionally, their frame partition scheme must be in accordance with the road layout that makes them depend on the road topology.

PTMAC [35] used a collision prediction-based mechanism to reduce packet collisions caused by the merging collision scenarios. A slot-merge collision between two vehicles that are three-hop apart is predicted using their two intermediate vehicles. The two intermediate vehicles can detect a common slot used by two different vehicles by examining the one-hop set. Intermediate vehicles then classify this detection as a possible collision based on the speeds and directions of susceptible vehicles. Then one of the vehicles is indicated to vacate and acquire a new time slot. However, the vacating vehicle, unaware of free slots within its three-hop range, can only select a new time slot based on its two-hop information, which can again lead to a similar collision situation. The problem becomes worse with the increasing vehicle density, as it is harder to obtain a new time slot that is free in its three-hop communication range. Furthermore, PTMAC uses RSU for its efficient operation in four-way road intersection scenarios.

In [36], the number of packet collisions due to merging collision scenarios is reduced by adjusting the transmission power, i.e., for higher vehicle density, the vehicles lower their transmission power to reduce their communication range and vice versa. However, the reduced communication range affects their surrounding awareness quality, which in turn affects vehicle safety. In [37], the received signal strength of neighboring vehicles is used for time slot management. In [38], a mechanism for adaptive beaconing is proposed for a distributed TDMA-based MAC protocol. Each vehicle calculates a coefficient, named the danger coefficient, based on the information received from neighboring vehicles. Then, each vehicle adjusts its beaconing rate based on the value of the danger coefficient. A greater value signifies a more dangerous situation, so the vehicle increases its beacon rate and vice versa. The protocols [39,40] mitigate packet collisions based on the motion prediction of vehicles. A comparison of distributed TDMA-based MAC protocols for VANETs can also be found in [41].

## 3. Preliminaries

### 3.1. Operation of a TDMA-Based MAC Protocol in VANETs

In VANETs, each vehicle is required to send a periodic message to update essential information to its neighboring vehicles and broadcast an emergency message as soon as possible in order to maintain road safety. In TDMA-based MAC protocol, each vehicle achieves this by using its owned time slot in each frame, which is acquired when joining the network.

The slot acquisition process is as follows. Each vehicle first listens to the channel for a time frame to determine the free time slots in the frame. It then randomly selects a free time slot to transmit. A free slot is the time slot that fulfils the following two conditions: (i) It is not in use by any other vehicle in the communication range of this vehicle; (ii) It does not cause the hidden terminal problem. This is achieved by selecting a time slot that is not owned by any other vehicle in the two-hop neighborhood [13,14,34]. If more than one vehicle in the two-hop neighborhood simultaneously selects the same free time slot for the transmission, packet collisions occur, which is called the access collision [8]. The vehicle must then repeat the aforementioned process until it is successful. However, once the vehicle successfully acquires a time slot, it keeps using this time slot until and unless it again loses the time slot.

**Figure 1:**
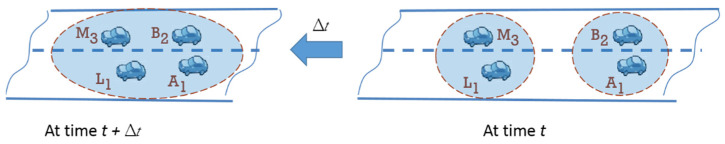
Example scenario of a merging collision.

A merging collision [8] can cause the vehicle to lose its owned time slot. Consider the merging collision scenario in Figure 1. At time *t*, vehicles *A* and *B* are within the communication range of each other and own the time slots 1 and 2, respectively. Similarly, vehicles *L* and *M* are within the communication range of each other and own the time slots 1 and 3. However, vehicles *A* and *B* are not within the communication range of vehicles *L* and *M*. Therefore, both *A* and *L* can transmit successfully despite possessing the same time slot, i.e., slot 1. After a time interval of ∆*t*, all four vehicles come within the communication range of each other. Now a packet collision will take place between the transmissions of *A* and *L*, and both their packets will be lost, i.e., *B* and *M* cannot receive the packets of *A* and *L*. Here, we say that *A* and *L* have met a slot-merge collision due to slot 1.

With the existing TDMA-based distributed MAC protocols for VANETs [13,27,28], both vehicles *A* and *L* lose their time slots in this particular scenario. Therefore, they need to acquire new time slots for their next transmissions. Now, if they select the same free time slot, an access collision occurs. We call this access collision the subsequent access collision because it occurs soon after the previous packet collision. A subsequent access collision means two or more successive packet collisions without a successful data transmission in between.

### 3.2. Extended Delay Problem

In general, the aftermath of a merging collision scenario can be following:

- Packets of vehicles whose transmissions met the packet collision are lost, and all such vehicles may need to acquire a new time slot for the next transmission.
- A single merging collision can spark subsequent access collisions.
- The above two consequences result in an extended delay in obtaining updates of the vehicles involved in the merging collision.

**Figure 2:**
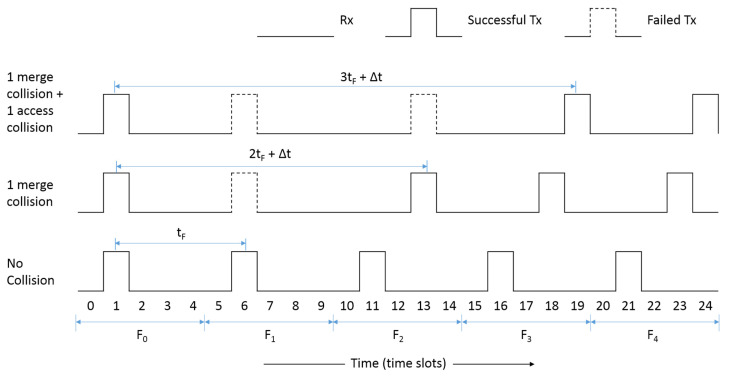
Transmission timelines for a typical node in a TDMA-based MAC for VANET representing some of the possible causes; the bottommost is a baseline case when no packet collision has occurred; the middle timeline represents the case when the vehicle met a slot-merge collision but no subsequent access collision and the uppermost represent the case when the vehicle met a slot-merge collision as well as a subsequent access collision thus causing the extended delay problem.

Consider the TDMA-based MAC environment shown in Figure 2, where each TDMA frame consists of a total number of five time slots, i.e., F_0_ is from *t* = 0 to *t* = 4, F_1_ is from *t* = 5 to *t* = 9, and so on. The figure shows the transmission timelines of a vehicle that initially owns the time slot 1 in F_0_. The bottom timeline is a case when the vehicle successfully transmits in each frame using its owned time slot 1 at *t* = 1, 6, 11, 16, 21 and there is no packet collision. Let *t*_F_ denote the length of a single TDMA frame, then the delay between the two consecutive transmissions of the vehicle is also *t*_F_.

Now consider the case in the middle timeline where the vehicle met a slot-merge collision in F_1_ at *t* = 6. As a result, the transmission of the vehicle in slot 1 of F_1_ was lost, and the vehicle lost its time slot. Therefore, the vehicle needed to reselect a new free time slot in F_2_ for its next transmission. As per the slot acquisition process, the vehicle listens to the channel until the cycle completes in time slot 1 of F_2_ and then selects one of the free time slots. However, up to this point, the time period 2*t*_F_ has been elapsed since the last successful transmission in slot 1 of F_0_. Assuming that time slot 3 is a free time slot, the vehicle selects time slot 3 as its new time slot in F_2_. An additional time period ∆*t*, which is less than *t*_F_, is also elapsed in F_2_ between slot 1 and slot 3. The transmissions through this time slot were successful, i.e., transmissions at *t* = 13, 18, 23. However, in this case, the delay between the first two successful transmissions, i.e., *t* = 1 to *t* = 13, is 2*t*_F_ + ∆*t*, as shown in the figure. Finally, consider the topmost timeline where the vehicle met the merging collision in F_1_ and then selected a new time slot 3 in F_2_ that met a subsequent access collision at *t* = 13. Therefore, the vehicle has to reselect a new time slot again in F_3_.

In F_3_, the vehicle selects time slot 4 through which transmissions were successful, i.e., at *t* = 19, 24. In this case, the delay between two successful transmissions, i.e., *t* = 1 to *t* = 19, is 3*t*_F_ + ∆*t*. This delay is greater than three times the regular time interval *t*_F_. Please note that it is never guaranteed that the vehicle will be successful after one subsequent access collision. The possibility of further access collisions remains, in which case the delay increases until the vehicle is successful in its transmission. In general, the delay *D* experienced by the vehicle after the merging collision can be given by:

*D* = *t*_F_ + *nt*_F_ + ∆*t* (1)

where *n* is the number of packet loss between two successful transmissions, *t*_F_ is the frame length and ∆*t* is the time elapsed in the frame in which the vehicle transmits successfully, i.e., between the time slot at which the cycle completes and the time slot at which the vehicle transmits.

The delay between two consecutive periodic messages is critical. A packet loss means an extended delay than the regular time interval. Moreover, the more subsequent access collisions, the more packet loss of vehicles occurs, resulting in the extended delay to prolong further, which we call the extended delay problem. From Equation (1), it is clear that the delay between two periodic messages is directly proportional to the number of packet losses. Therefore, our research aims to minimize *n* by avoiding the subsequent access collisions in order to restrict the extended delay from prolonging.

## 4. MCCM-MAC Protocol

MCCM-MAC is a distributed TDMA-based MAC protocol for VANETs. The slot management is not controlled by any central entity. Each vehicle is responsible for its own time slot acquisition and vacation. For this purpose, each vehicle maintains the currently-known vehicles set (CVS) and the previously-known vehicles set (PVS) and exchanges these sets with the neighboring vehicles. Using these sets, each vehicle detects the slot-merge collision of its own slot as well as the slot-merge collision of other neighboring vehicles. The relevant mechanisms are referred to as the self slot-merge collision and the third parties slot-merge collision, respectively. The packet collisions are then mitigated through the third parties slot-merge collision mechanism, slot suggestion mechanism, and tie breakup mechanism. The basic principle is to avoid a subsequent access collision after a packet collision which not only reduces the overall packet loss count but also avoids the prolonged delays between two consecutive periodic messages.

**Figure 3:**
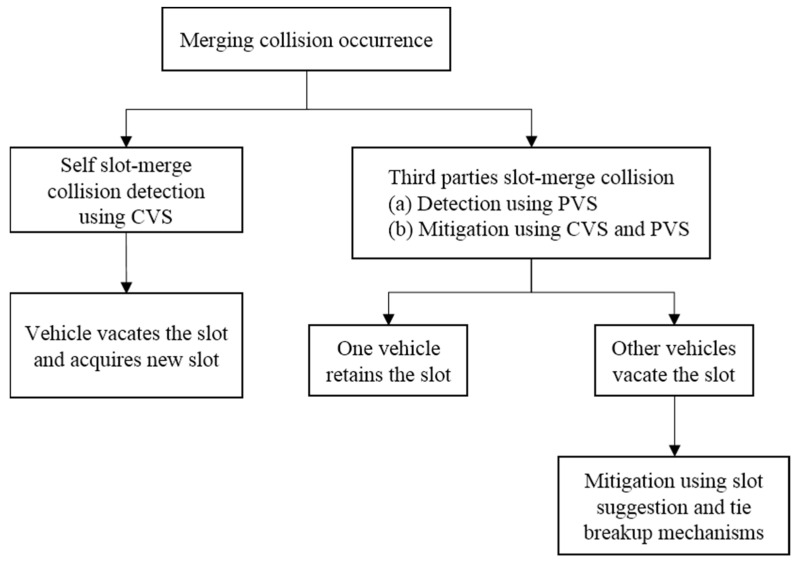
Merging collision mitigation in MCCM-MAC protocol.

The process for mitigating the merging collision scenario is shown in Figure 3. On the occurrence of a merging collision between the vehicles, their common neighboring vehicles can detect the merging collision through third parties slot-merge collision and mitigate it by indicating one of the vehicles in the slot-merge collision to retain the time slot. The other vehicles in the slot-merge collision detect the self slot-merge collision and vacate their time slots. The vacating vehicles can receive a suggestion for selecting a new time slot from a known neighboring vehicle through the slot suggestion mechanism. However, in case a packet collision cannot be avoided, it does occur. Then the neighboring vehicles, through the tie-breakup mechanism, indicate one of the vehicles in the packet collision to retain the time slot and others to vacate it. This can avoid further packet collisions.

In general, MCCM-MAC can avoid another packet collision after a packet collision by employing the proposed mechanisms. This enables a particular vehicle to avoid successive packet collisions, thereby avoiding the prolonged delay between its periodic messages.

### 4.1. CVS and PVS Formation

Each vehicle has a unique vehicle ID [13,14]. It must also possess a time slot in the frame for transmission. Therefore, the transmission of a vehicle can be characterized by an ordered pair (VehicleID, SlotNo). Each vehicle maintains the following two sets locally. The vehicle updates these sets in every time slot and then broadcasts the latest in its periodic message.

**Figure 4:**
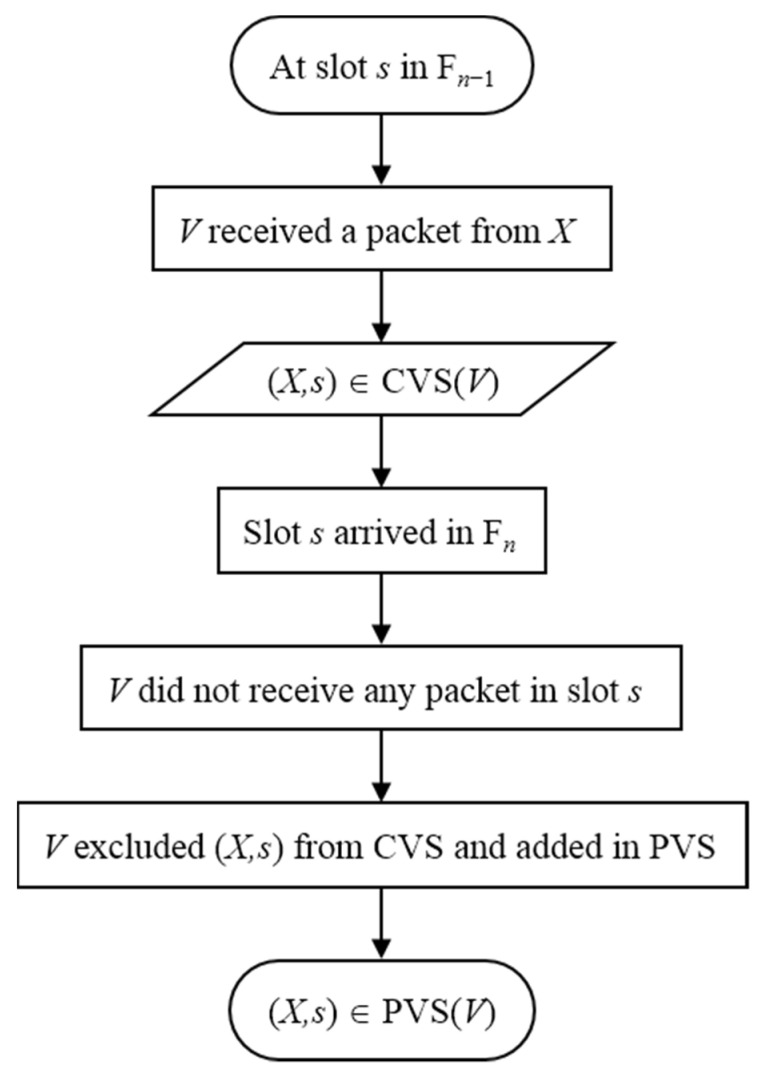
Procedure for adding an entry in the PVS set.

- Currently-known vehicles set (CVS): After every successful reception, the vehicle records an ordered pair consisting of the source vehicles’ ID and slot number in its CVS, i.e., (srcVehicleID, srcSlotNo) ∈ CVS. This set constitutes the known vehicles that are directly within the communication range of this vehicle and possess a valid time slot.
- Previously-known vehicles set (PVS): Let F*_n_* represent the current frame cycle, and F*_n_*_−1_ represent the previous frame cycle. Vehicle *V* has an entry (*X*,*s*) ∈ CVS in the frame cycle F*_n_*_−1,_ but *V* did not have any successful reception in the time slot *s* of the next frame cycle F*_n_* then (*X*,*s*) ∈ PVS. This procedure is also depicted in Figure 4. This set constitutes the vehicles that might have possibly met the merging collision.

### 4.2. Known and Unknown Vehicles

At the point of receiving a packet from a source vehicle in F*_n_*, the vehicle classifies the source vehicle as a known vehicle or an unknown vehicle. If the vehicle has already listened to the same source in the previous frame, i.e., in F*_n_*_−1_ (srcVehicleID, srcSlotNo) ∈ CVS, then the source vehicle is classified as a known vehicle. However, if the vehicle has not listened to it in the previous frame, i.e., in F*_n_*_−1_ (srcVehicleID, srcSlotNo) ∉ CVS, then the source vehicle is classified as an unknown vehicle. This classification is useful in detecting and mitigating packet collisions, as discussed in the next sections.

### 4.3. Self Slot-Merge Collision

Each vehicle checks for the status of its owned time slot on every reception. If the vehicle detects a slot lost at any point, then it needs to acquire a new time slot. A vehicle loses its time slot if it finds that a packet is from a known vehicle, but the received CVS does not include its entry. With an ideal channel, this condition can only be true in the case of a merging collision [13,14]. Let vehicle *V* own time slot *t_v_*, and *V* receives a packet from a source vehicle *Y* in the current time slot *t_c_*. If *Y* is an unknown vehicle, then (*Y*,*t_c_*) is added in CVS(*V*), but the lost slot condition is not checked in this case. However, if *Y* is a known vehicle, then the following lost slot condition is checked. If (*V*,*t_v_*) ∈ CVS(*Y*) then *V* retains the time slot *t_v_*, but if (*V*,*t_v_*) ∉ CVS(*Y*), then *V* has met a slot-merge collision and lost its time slot *t_v_*. The aforementioned procedure for detecting the self slot-merge collision is compatible with the VeMAC protocol and takes into consideration its slot release prevention condition [13]. However, we complement it with the proposed third parties slot-merge collision procedure.

### 4.4. Third Parties Slot-Merge Collision

**Figure 5:**
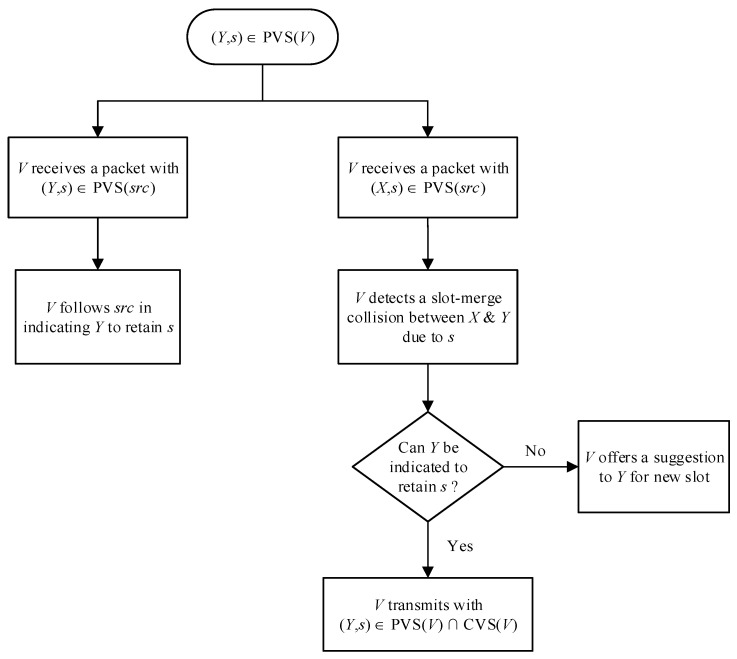
Overview of the third parties slot-merge collision mechanism.

In addition to detecting the slot-merge collision for its own time slot, we propose that each vehicle also detects any other merging collision which has taken place within its communication range. We call this third parties slot-merge collision detection because this vehicle is not itself part of the merging collision. On detection of such a merging collision, we mitigate it by ensuring that one vehicle keeps using the time slot and other vehicles vacate it. This is achieved using the following rules on the reception of each packet. The overview of these rules is also given in the Figure 5.

Rule I. The vehicle *V* detects a slot-merge collision between two other vehicles, *X* and *Y* if (*Y*,*s*) ∈ PVS(*V*) and *V* receives a packet from a source such that (*X*,*s*) ∈ PVS(*src*) where *X* and *Y* are third party vehicles which possess the same slot *s*. Here *X* ≠ *Y* ≠ *V* ≠ *src*.

Rule II-a. If the vehicle *V* with (*Y*,*s*) ∈ *PVS* has detected the slot-merge collision between the two third party vehicles *X* and *Y*, then *V* indicates vehicle *Y* to retain the slot by including (*Y*,*s*) in both sets CVS and PVS, i.e., (*Y*,*s*) ∈ PVS(*V*), (*Y*,*s*) ∈ CVS(*V*).

Rule II-b. If the vehicle *V* with (*Y*,*s*) ∈ PVS has detected the slot-merge collision between the two third party vehicles *X* and *Y*, but with (*X*,*s*) ∈ PVS(*src*) and (*X*,*s*) ∈ CVS(*src*)*,* then it means the source vehicle has already indicated *X* to retain the slot *s*, therefore, *V* does not indicate vehicle *Y* to retain the slot *s* and thus does not include (*Y*,*s*) in its CVS, i.e., (*Y*,*s*) ∈ PVS(*V*), (*Y*,*s*) ∉ CVS(*V*).

Rule III. If the vehicle *V* with (*Y*,*s*) ∈ PVS has detected the slot-merge collision between two third party vehicles *X* and *Y* but cannot indicate vehicle *Y* to retain the slot (due to Rule II-b), then it offers a suggestion of a possible free slot for *Y* to select as a new slot.

Rule IV. If vehicle *V* has received a packet from a source vehicle with exactly the same entry in PVS, i.e., (*Y*,*s*) ∈ PVS(*V*), (*Y*,*s*) ∈ PVS(*src*), then the source vehicle takes the lead role to indicate *Y* whether to retain the slot *s* or not, and the vehicle *V* adapts according to the source vehicle, i.e., *if* (*Y*,*s*) ∈ CVS(*src*) then (*Y*,*s*) ∈ CVS(*V*) and *if* (*Y*,*s*) ∉ CVS(*src*) then (*Y*,*s*) ∉ CVS(*V*), and *V* does not change this state further.

### 4.5. Slot Suggestion Mechanism

If the vehicle, which has identified a third parties merging collision, cannot indicate its known vehicle in the merging collision to retain the time slot, then it offers a slot suggestion for it to acquire a new time slot. The vehicle selects a free slot for its known vehicle and broadcasts in its periodic message. All other surrounding vehicles are informed of the status of this time slot and do not select it as a free slot if possible. This reduces the chance of packet collision if this free slot is selected for transmission.

**Figure 6:**
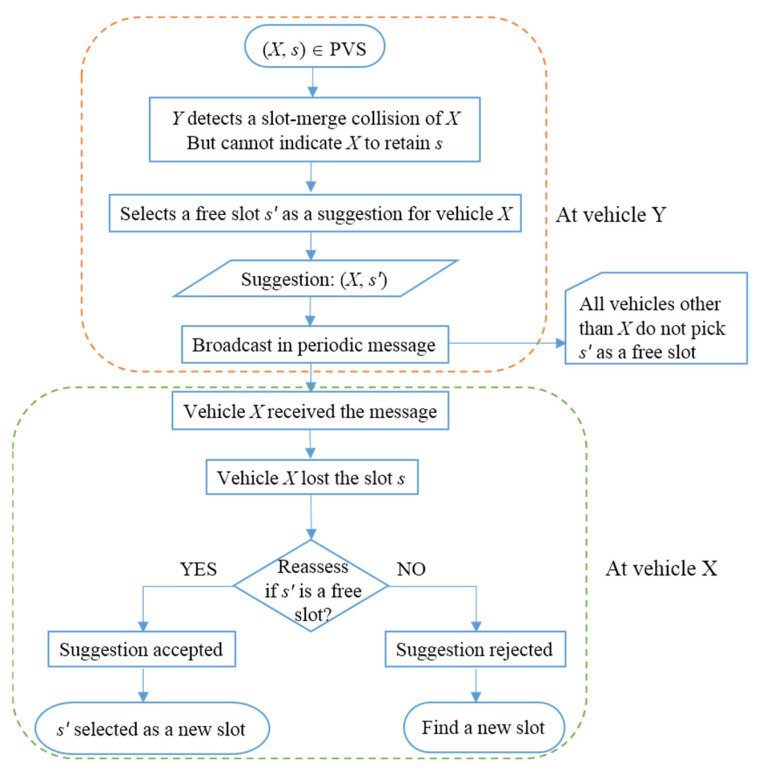
Vehicle *Y* suggests a slot to vehicle *X*.

Suppose *X* and *Y* are known vehicles to each other in frame F*_n_* and own slots *s* and *r,* respectively, i.e., (*X*,*s*) ∈ CVS(*Y*) and (*Y*,*r*) ∈ CVS(*X*). This means both were able to successfully listen to each other’s packet in frame F*_n_*. In the next frame, F*_n_*_+1_, however, *X* met a slot merge-collision, which was detected by *Y* (with the help of Rules I, II and III), but *Y* cannot indicate *X* to retain the slot. Now, *Y* transmits its periodic message with (*X*,*s*) ∈ PVS(*Y*) and *s* as a suggestion slot for *X*. On receiving this message, all vehicles become aware of slot *s*. All vehicles other than *X* do not pick *s* as a free slot as long as any other slot is available for them. The vehicle *X* reassesses the suggested slot *s* and if it is a valid free slot, then the vehicle selects it; otherwise, it selects another new slot. This process is also depicted in Figure 6.

### 4.6. Example Scenario

**Figure 7:**
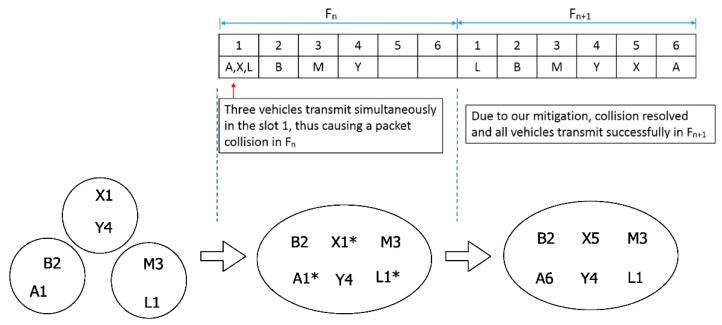
A merging collision scenario between three vehicles A, L and X; L retains the time slot despite its slot-merge collision due to the third parties slot-merge collision detection; A and X avoid the subsequent access collision due to the slot suggestion mechanism. * indicates the slot-merge collision.

We now demonstrate the third parties slot-merge collision and slot suggestion mechanisms through an example where a slot-merge collision between three vehicles occurs. Consider the example scenario shown in Figure 7, G_1_ = {(A,1), (B,2)} = CVS(A) = CVS(B), G_2_ = {(L,1), (M,3)} = CVS(L) = CVS(M) and G_3_ = {(X,1), (Y,4)} = CVS (X) = CVS(Y) where (*N*, *s*) means the vehicle *N* owns the time slot *s*. Initially at time *T*, G_1_, G_2_ and G_3_ are out of range from each other, therefore there is no interference due to s(A) = s(X) = s(L) = 1 where s(N) represents the time slot of vehicle *N*. However, at time *T* + ∆*t* all the groups G_1_, G_2_ and G_3_ have merged such that all vehicles are now within range of each other.

Now in slot 1 of F*_n_*, all three vehicles A, L and X transmit, resulting in a packet collision and hence the packets of these vehicles are lost. As per our protocol, vehicle B adds (A,1) to its PVS, vehicle M adds (L,1) to its PVS and vehicle Y adds (X,1) to its PVS (recap the procedure for adding an entry in PVS in Section 4.1).

In slot 2, B transmits, and all others receive its message successfully; vehicle A detects the lost slot for its owned slot and vacates slot 1 (Section 4.3); vehicle M detects a third parties slot-merge collision between vehicles L and A with the help of proposed Rule I (Section 4.4); similarly, vehicle Y detects the slot-merge collision between vehicles X and A. In slot 3, as per Rule II-a, vehicle M transmits its message with (L,1) belonging to its CVS and PVS, which all others receive successfully. The transmission of M included (L,1) ∈ CVS, therefore, as per the lost slot detection algorithm, L does not vacate slot 1 and retains it. Meanwhile, vehicle Y detects that L has been indicated by M to retain slot 1, so Y does not indicate vehicle X to retain slot 1 as per Rule II-b. However, Y includes a suggestion for X for a free slot, e.g., slot 5, as per Rule III. In slot 4, Y transmits, which leads to the lost slot at X because (X,1) ∉ CVS(Y), so vehicle X vacates slot 1. However, X has a suggestion for a new slot, i.e., slot 5, and vehicle A also knows about this suggestion.

In the next frame, F*_n_*_+1_ at slot 1, there are two candidates, i.e., vehicle A and X, which need to acquire new slots as both have lost their slots in the previous frame F*_n_*. Vehicle *A*, being aware of the suggestion for X, will not choose slot 5 as a new slot; therefore, it selects any other free slot, i.e., slot 6. Vehicle X will reassess the received suggestion, find that slot 5 is free, and select slot 5 as the new slot. This eliminates the competition between A and X enabling both to acquire the slots successfully.

In short, a packet collision between the transmissions of A, L and X took place in frame F*_n_*_,_ and all three vehicles were again able to successfully transmit in the next frame F*_n_*_+1_ avoiding the extended delay problem.

### 4.7. Tie-Breakup Mechanism

If multiple vehicles, which lost a slot due to the merging collision, happen to select the same time slot again, then a subsequent access collision takes place. To avoid further subsequent access collisions, we employ the tie-breakup mechanism to mitigate it such that one of the vehicles keeps using this slot, and the others vacate the slot. The vehicle that vacates has to acquire a new time slot in the next frame, but its competitor would not be there as it retained the old slot. In this way, another subsequent access collision is avoided.

To achieve the above, we propose that whenever a vehicle selects a new slot, it sets a special message which indicates that this is the first transmission through this time slot. It is essentially the same periodic message but only with a special feature set. Hereafter, we refer to this transmission as the first transmission (FT). In addition, a vehicle can distinguish the FT entries in CVS. Then the following rules are used for the mitigation:

Rule V. If the vehicle *V* receives a packet from a source with an FT entry (*X*,*s*) ∈ CVS(*src*) and there is no entry against time slot *s* in its own CVS, i.e., *s* ∉ CVS(*V*), then *V* adds this received entry into its CVS. However, these FT entries cannot be used for PVS calculation.

Rule VI. If the vehicle *V* receives an FT entry against a time slot for which *V* already has an FT entry in its CVS, then *V* retains the entry of the lowest vehicle ID and the entry of higher vehicle ID is discarded.

**Figure 8:**
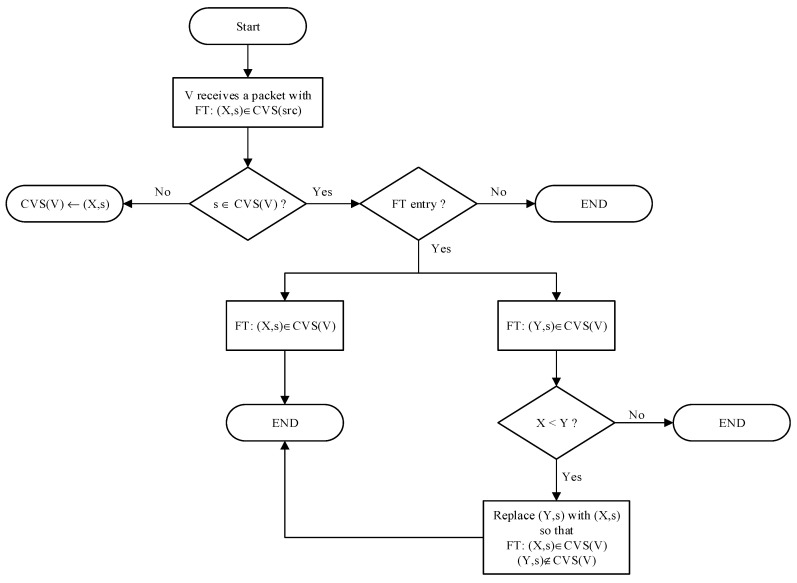
Overview of the tie-breakup mechanism.

A flow chart of the above rules is given in Figure 8. In Rule VI, vehicle ID is used to break the tie between two FT entries. The entry with the lowest vehicle ID has no special significance, but we need to define a criterion to retain one entry and discard another. Another important point is that the entries added in CVS by virtue of Rule V are those entries from which the vehicle has not listened directly; therefore, these entries do not qualify to be added in PVS. Ignoring this important point will lead to the malfunction of third parties slot-merge collision detection and hence degradation of the performance.

**Figure 9:**
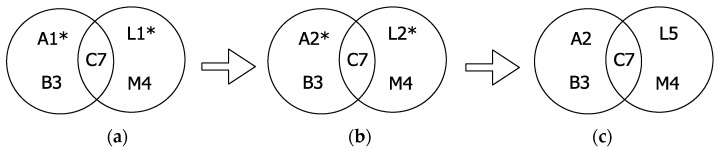
Example scenario for the tie breakup mechanism. (**a**) slot-merge collision between A and L in time slot 1 at frame F*_n_*; (**b**) Subsequent access collision between A and L in time slot 2 at frame F*_n_*_+1_; (**c**) Tie breakup mechanism enables A to retain time slot 2 and L acquires a new time slot 5 in F*_n_*_+2_. * indicates the time slots that caused the packet collisions.

We now demonstrate the tie-breakup mechanism through an example scenario shown in Figure 9. Let the vehicles merge as per the topology shown in Figure 9a in time slot 6 of F*_n_*_−1_. This leads to a slot-merge collision between vehicle A and L in time slot 1 of F*_n_*, which is experienced by vehicle C. Here vehicles B and M received the same packet successfully, which was lost at vehicle C, and thus were not able to detect the packet collision through third parties slot-merge collision mechanism. As soon as C transmits, both the vehicles A and L lose their slots in frame F*_n_*. Therefore, both vehicles need to acquire new slots for their next transmission. Considering the case that vehicle A and L selected the same time slot once again in the next frame, F*_n_*_+1_, which can never be ruled out, say slot 2 as a new time slot for their next transmissions. Therefore, packet collision occurs again at C in time slot 2, and C cannot receive any transmission in this slot, so slot 2 ∉ CVS(C). However, despite the packet loss at C, vehicle B was able to receive the FT from A, and likewise, M received the FT from L. In slot 3, C finds the FT entry of A in CVS of B, i.e., (A,2) ∈ CVS(B), and that slot 2 is a non-occupied slot in its own CVS, i.e., slot 2 ∉ CVS(C); therefore as per Rule V, the vehicle C adds this entry into its CVS, i.e., CVS(C) ← (A,2). In slot 4, vehicle C received another FT entry (L,2) ∈ CVS(M), whereas it already had an FT entry for slot 2. Now, as per Rule VI, vehicle C prefers the entry with the lowest vehicle id. Assuming that vehicle A has a lower vehicle ID than L, then (A,2) will be kept and (L,2) will be discarded. In slot 7, C transmits with (A,2) ∈ CVS(C); on reception from C the vehicle A retains slot 2, L vacates it and M updates the FT entry (A,2). In the next frame, F*_n_*_+2_, A transmits successfully through slot 2, and L selects a new slot from the free slots. In this example scenario, vehicle C has broken the tie between A and L successfully.

### 4.8. Packet Structure

**Figure 10:**
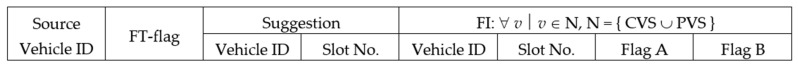
Packet structure of a periodic message in MCCM-MAC.

The packet structure of a periodic message in the MCCM-MAC protocol is shown in Figure 10. The first field *Source vehicle ID* is the vehicle ID of the packet sender. The *FT-flag* is a single bit that is set when the vehicle is sending its first transmission through a newly selected time slot. It is used in the tie-breakup mechanism. The *suggestion* field is used if a vehicle wants to propose a free slot to another vehicle. Currently, we have limited one vehicle to suggest a slot for only one other vehicle within one frame. In a TDMA-based MAC protocol, every vehicle sends its one-hop neighbor’s information, i.e., <vehicleID, slotNo>, in its periodic message. This is also known as frame information (FI). MCCM-MAC needs two additional bits with each entry in FI, i.e., flags A and B. The sending vehicle indicates the status of each entry by setting these flags according to Table 1.

## 5. Simulation Results

We have performed extensive simulations to compare the performance of our proposed protocol. In experiment 1, we study the performance in highway and city scenarios. Whereas, with experiment 2, we study the effect of the slot-merge collision between two vehicles (mc-2) and the slot-merge collision between three vehicles (mc-3) in an isolated environment. 

The simulation environment consists of the Open Modular Network Testbed in C++ (OMNET++) version 5.4.1 [42,43]. Using INET framework version 3.6.4 [44,45] for OMNET++, we have implemented three MAC modules corresponding to the VeMAC, MAC-AC and MCCM-MAC protocols. The INET mobility models are used for simulating vehicle mobility. In the highway scenario, a linear mobility model is used, and in the city scenario, a combination of rectangular and tractor mobility models are used.

### 5.1. Experiment 1

In this experiment, we simulate the performance of three protocols, VeMAC [13], MAC-AC [28] and MCCM-MAC, in a highway and a city scenario. Three performance metrics are considered: packet loss count, average delay and packet delivery ratio. Each packet loss indicates an instance of the extended delay. The lower packet loss count signifies fewer instances of prolonged delay. Thus, the greater packet loss count signifies poorer performance. The delay represents the time elapsed between two consecutive successfully delivered periodic messages for a particular vehicle. It measures the capability of the protocol for time-bound message delivery. Decreased delay signifies better performance. In an ideal condition, when there is no packet loss, the average delay is equal to frame length, i.e., 100 ms for our experiment. Several traffic safety applications require it to be 100 ms. This is why a typical size of the TDMA frame is taken as 100 ms in the majority of the existing TDMA MAC protocols for VANETs [46]. However, with packet loss, the average delay increases beyond 100 ms. Together, packet loss count and average delay can describe the protocol’s suitability for the time-bound message delivery. Packet delivery ratio (PDR) is the ratio of the number of transmissions successfully delivered to the ideal number of transmissions. Ideally, each vehicle must have delivered a periodic message in each frame. If transmissions of all vehicles are delivered successfully in each frame, then PDR is equal to one; if all transmissions are lost, PDR is zero.

**Figure 11:**
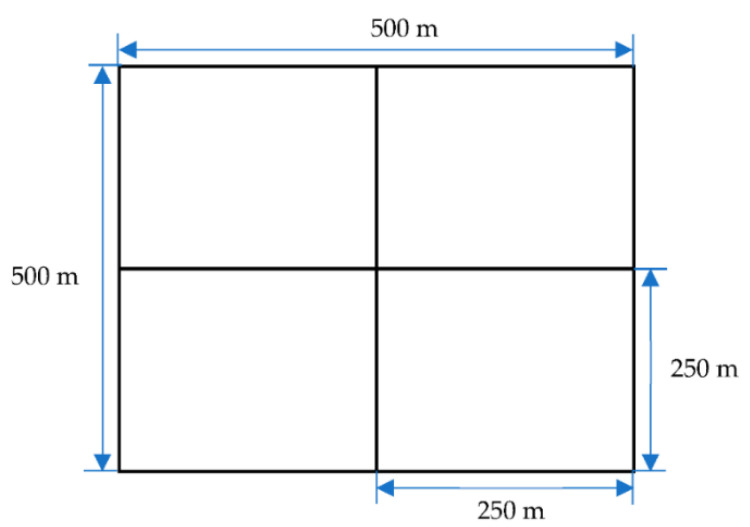
The layout of the city scenario.

A two-way highway of 1000 m is considered for a highway scenario on which vehicles can travel in both directions. For a city scenario, we have considered six streets, each of 500 m, connected forming four corners, four three-way intersections, and one four-way intersection. The road topology for the city scenario is shown in Figure 11. Initially, all vehicles in the experiment possess a valid slot through which vehicles can transmit successfully without causing any packet collision. However, as the simulation progresses, vehicles may meet the merging collision scenario due to vehicle mobility, resulting in the packet loss count. The channel is ideal; therefore, all the packet loss is either due to merging collisions or subsequent access collisions, which cause the vehicle to experience an extended delay. The number of vehicles is varied for each simulation run, but it remains fixed during a particular simulation run. The total number of slots in the TDMA frame remains fixed for all simulation runs.

All protocols are simulated without any frame partitioning because our main purpose is to evaluate the performance of a protocol without frame partitioning. The version of VeMAC without disjoint partition is considered, i.e., “VeMAC with τ = 0” [13]. MAC-AC [28] is given the maximum leverage by assigning a unique “competition timestamp” [28] for each vehicle, which is the best case for MAC-AC. The rest of the simulation parameters are given in Table 2.

**Figure 12:**
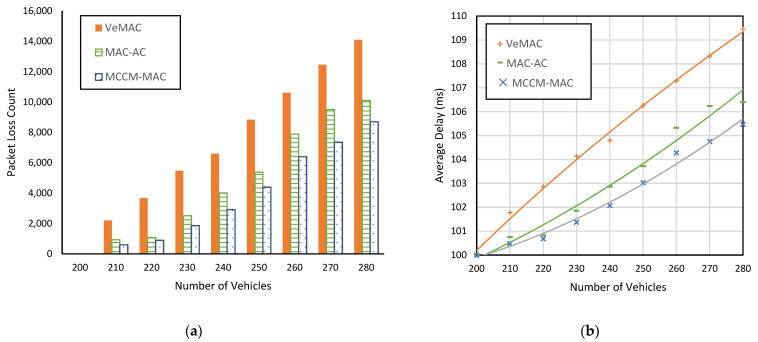
Comparison of the protocols in a highway scenario with different vehicle densities, (**a**) packet loss count of periodic messages, (**b**) average delay between the successfully delivered periodic messages.

**Figure 13:**
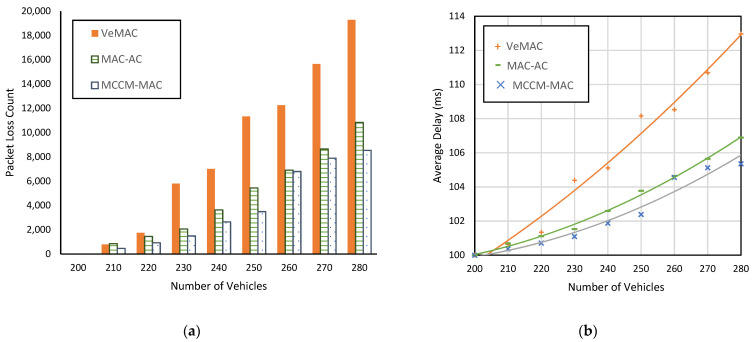
Comparison of the protocols in a city scenario with different vehicle densities, (**a**) packet loss count of periodic messages, (**b**) average delay between the successfully delivered periodic messages.

Let *Nv* represent the total number of vehicles in a simulation run, and *Ns* be the number of time slots in a frame. In the first simulation run for both scenarios, *Nv* = 200, where each vehicle possesses a unique slot. As shown in Figure 12a and Figure 13a, no collision is recorded for this scenario with all protocols. This is because, for *Nv* ≤ *Ns*, if each vehicle possesses a unique slot, no merging collision is possible. However, as we repeat the experiment with more vehicles, i.e., *Nv* > 200, the collision count gradually increases. This is because for *Nv* > *Ns*, not every vehicle can possess a unique slot, and due to the vehicle mobility, whenever a merging collision scenario is encountered, packet collisions occur.

Figure 12 and Figure 13 show the effect of varying vehicle density on the packet loss incurred and the average delay between two consecutive successful periodic messages. It can be seen in Figure 12a and Figure 13a that our proposed protocol MCCM-MAC always remains lowerin packet lost count than VeMAC and MAC-AC. For example, in a highway scenario with a vehicle count of 230, MCCM-MAC resulted in 66% fewer packet loss counts than VeMAC and 26% fewer than MAC-AC. In a city scenario, MCCM-MAC reduced packet loss up to 74% (i.e., for *Nv* = 230) over VeMAC and up to 35% (i.e., for *Nv* = 250) over MAC-AC. On average, MCCM-MAC improved 55% over VeMAC and 23% over MAC-AC in terms of packet loss count.

Similarly, it can be seen in Figure 12b and Figure 13b that MCCM-MAC resulted in a lower delay than VeMAC and MAC-AC, which demonstrates its effectiveness for time-bound message delivery. For example, in a city scenario with 280 vehicles, the average delay between two consecutive periodic messages was 113 ms with VeMAC and 107 ms with MAC-AC, whereas with MCCM-MAC, it was only 105 ms. 

**Figure 14:**
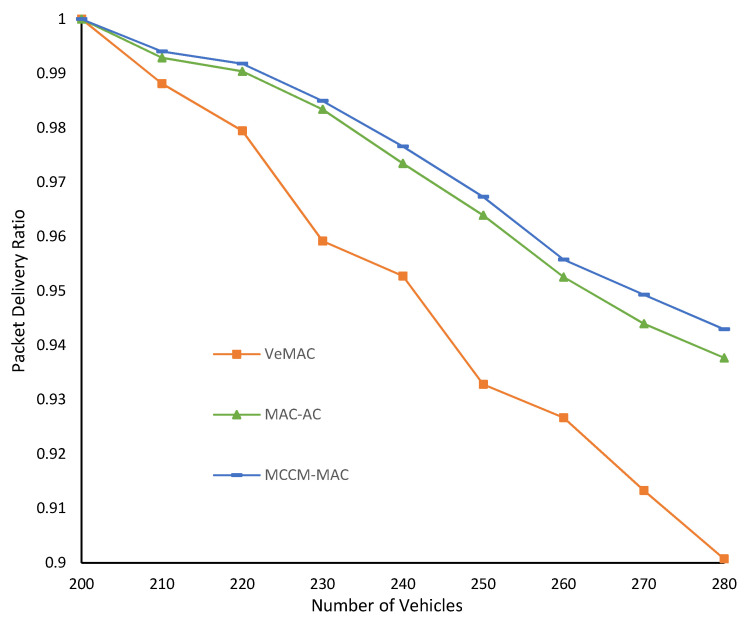
Average packet delivery ratio.

Figure 14 show the packet delivery ratio (PDR). Each value is the average across both scenarios for a particular number of vehicles. MCCM-MAC remained higher than VeMAC and MAC-AC throughout for all vehicle densities. Even for a high-density environment with *Nv* = 280, the PDR of MCCM-MAC was above 94%. On average, MCCM-MAC delivered the periodic messages with 97% PDR, which was the highest among competitors, and with an average delay of 102 ms, which was the lowest among others.

In general, as the vehicle density increased, the performance of all protocols degraded. This is because, with the greater vehicle density, there is a decreased number of available free slots. The lower the number of free slots, the greater the chance of packet collisions. However, the proposed protocol MCCM-MAC remained superior to the VeMAC protocol and MAC-AC protocol for both highway and city scenarios in terms of packet loss count, delay, and packet delivery ratio. The reason is that our mitigation of the merging collision results in decreased slot loss and hence reduced subsequent access collisions. In VeMAC, most of the merging collision scenarios result in the slot loss of both vehicles, causing a greater chance for a subsequent access collision. This subsequent access collision can then also lead to another subsequent access collision. Therefore, the packet loss in VeMAC is the highest.

In MAC-AC, there is no measure to avoid a subsequent access collision soon after the merging collision. However, after the first subsequent access collision, it is tried that one vehicle keeps using the same old slot. Therefore, the packet loss in MAC-AC is less than in VeMAC. It must also be pertinent that the results shown for MAC-AC represent the best case of MAC-AC because we have assigned a unique competition timestamp for each vehicle. The limitation of MAC-AC is that the access collision between transmissions of two vehicles can be mitigated only if they have different competition timestamps. The competition timestamp of the vehicle is the time at which it receives the first packet. In reality, there is always a possibility that vehicles can have the same competition timestamp, in which case the MAC performance degrades. In the worst case of MAC-AC, when each vehicle has the same competition timestamp so access collisions cannot be mitigated, the performance of MAC-AC approaches VeMAC. Nevertheless, MCCM-MAC does not suffer from limitation such as MAC-AC. Moreover, MCCM-MAC mitigates both the merging collisions and the subsequent access collisions, resulting in the lowest packet loss count. In the instances of merging collision where mc-2 and mc-3 took place as per scenarios studied in experiment 2, MCCM-MAC can avoid the subsequent access collision, whereas VeMAC and MAC-AC cannot do so. Consequently, MCCM-MAC remained superior in both highway and city scenarios.

### 5.2. Experiment 2

In this experiment, we particularly focus on the aftermath of the merging collisions through some particular simulation scenarios in an isolated environment. The packet loss ratio is evaluated for the vehicles that meet the slot-merge collision. It is the ratio of the number of packets lost to the number of packets transmitted by those vehicles. In the absence of any packet loss, this ratio is zero; if all packets are lost, then the ratio is one. The lesser value signifies better performance, i.e., fewer subsequent access collisions and less extended delay.

**Figure 15:**
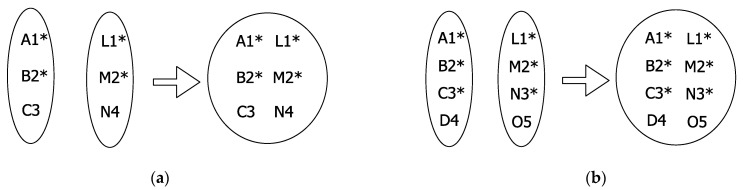
Example for (**a**) Simulation scenario #2 where two mc-2 take place, i.e. between nodes (i) A and L, (ii) B and M; (**b**) Simulation scenario #3 where three mc-2 take place, i.e. between nodes (i) A and L, (ii) B and M, (iii) C and N; * shows the nodes that meet the slot-merge collision, mc-2 represents a slot-merge collision between two nodes.

Four simulation scenarios are considered. In simulation scenario #1, the effect of one slot-merge collision between two vehicles (mc-2) is studied. Two sets of vehicles are considered, as shown in Figure 1. There are only two vehicles, one in each set, which possess the same time slot. However, initially, this does not cause communication interference because both vehicles are far enough to avoid communication interference. Therefore, in the first frame, F_0_, all transmissions are successful. In the next frame, F_1_, both sets are merged, and one slot-merge collision between two vehicles (mc-2) occurs. Simulation is run for two more frames, and results are recorded for F_1_ to F_3_. In simulation scenario #2 (Figure 15a), two slots are common in each set such that two slot-merge collisions (mc-2) take place in F_1_. Similarly, in simulation scenario #3 (Figure 15b), three slots are common in each set such that three slot-merge collisions (mc-2) take place in F_1_. However, in simulation scenario #4 (Figure 7), three sets are considered with one slot common resulting in a slot-merge collision between three vehicles (mc-3).

**Figure 16:**
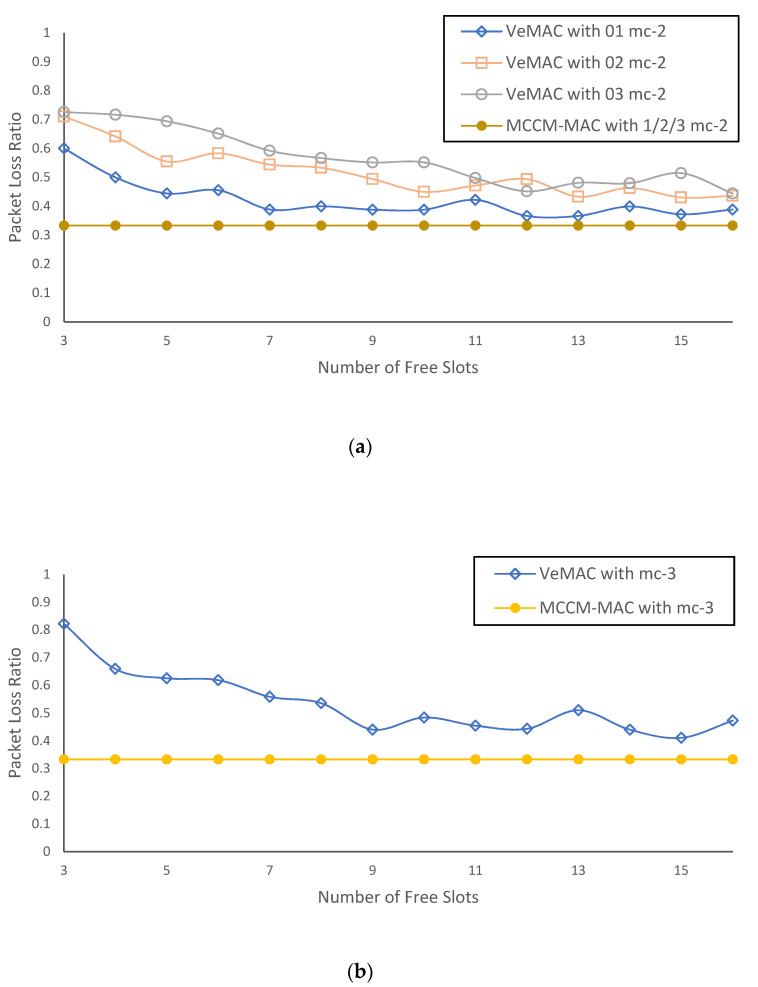
The packet loss ratio (PLR) of vehicles that suffered a slot-merge collision in the aftermath of (**a**) merging collisions between two vehicles (mc-2), (**b**) a merging collision between three vehicles (mc-3). The proposed protocol remains lower in PLR and is unaffected with the increase in vehicle density in the two-hop neighborhood as long as the minimum required number of free slots are available, i.e., free slots count ≥ vehicles count which compete for acquiring a new slot.

Figure 16 compare the packet loss ratio of the vehicles that met the slot-merge collision in the aforementioned scenarios with VeMAC [13] and MCCM-MAC protocols. The X-axis shows the number of available free time slots at the time of the merging collisions. The number of free time slots is inversely proportional to the vehicle density in the two-hop neighborhood of the vehicle. A lesser number of free slots signify greater vehicle density. The Y-axis shows the corresponding packet loss ratio. Each data point represents the average value of 30 repetitions. The graph of the VeMAC protocol shows that the higher the vehicle density, the more packet loss is results from a merging collision. This is because when a merging collision takes place between two vehicles, both vehicles lose their respective slots and they have to randomly pick a new slot from the available set of free slots. If there are fewer available free slots, then there are greater chances that both vehicles will select the same time slot again.

In contrast, the graph of the MCCM-MAC protocol shows that the packet loss ratio of the vehicles which met the merging collision is independent of available free slots as long as at least one free slot is available for each vacating vehicle. Furthermore, it remains lower in the packet loss ratio than the VeMAC protocol. This is because after the merging collision between two vehicles (mc-2) in the given scenarios, one of the vehicles retains the existing slot, and the other one picks the new slot. As there was only one vehicle that needed to acquire the new slot, there was no competition and hence no packet collision chance. If the merge collision was between three vehicles (mc-3), then one of the vehicles kept using the existing slot due to our mitigation of third parties slot-merge collision, and others acquired the new slots through the slot suggestion mechanism, thereby avoiding the packet collision. 

In general, the results of this experiment demonstrated the effectiveness of our mitigation of the slot-merge collision, which has resulted in a lower packet loss ratio for the vehicles that met the packet collision due to the merging collision scenario. The higher packet loss indicates more subsequent access collisions and the extended delay problem. The existing protocols cannot mitigate in these particular scenarios causing all the vehicles which have met mc-2 or mc-3 to vacate their existing slot. The occurrence of these merging collision scenarios in the highway and city environment causes deterioration in the protocol’s performance, which is also evident from the results of experiment 1.

## 6. Conclusions and Future Work

This paper has presented a distributed TDMA-based MAC protocol that mitigates packet collisions by improving their aftermath scenario. Using three novel mechanisms, i.e., the third parties slot-merge collision mechanism, slot suggestion mechanism and tie breakup mechanism, the proposed protocol enables one of the vehicles in the packet collision to retain the slot, which leads to two-fold benefits: early recovery of vehicles after the packet collision and reduced subsequent access collisions. Our simulation results show that the proposed protocol significantly reduced the number of packet losses, i.e., 55% fewer than VeMAC and 23% fewer than MAC-AC. On average, MCCM-MAC delivered the periodic messages with 97% PDR, which was the highest among competitors, and with an average delay of 102 ms between the successful periodic messages, which was the lowest among others.

In the future, we intend to study the impact of poor channel conditions on the performance of these protocols. We further intend to develop a machine-learning-based solution to detect the packet collisions in a distributed TDMA-based MAC protocol for VANETs.

## Figures and Tables

**Table 1:** FI entry’s classification based on Flag A and Flag B.

Flag A	Flag B	Classification
0	0	CVS
0	1	CVS + FT
1	0	PVS
1	1	PVS + CVS

**Table 2:** The simulation parameters.

Parameter	Value
Frame size	100 ms
Slot size	0.5 ms
Number of slots in a frame	200
Number of vehicles	200–280
Speed of vehicles	80–120 km/h (uniform distribution)
Transmission range	200 m
Data rate	6 Mbps
Simulation duration	600 frames
Mobility framework	INET [44,45]
